# Longitudinal assessment of preparation for care transition among adolescents and young adults with rheumatologic disease: a single-center pilot study

**DOI:** 10.1186/s12969-022-00751-1

**Published:** 2022-10-21

**Authors:** Jordan E. Roberts, Olha Halyabar, Carter R. Petty, Maria Alfieri, Brittany Esty, Johnathan Dallas, Melissa Hazen, Sandra Stein, Mary Beth F. Son

**Affiliations:** 1 Division of Rheumatology, Seattle Children’s Hospital/Seattle Children’s Research Institute, University of Washington School of Medicine, 4800 Sand Point Way NE, Mail Stop MA.7.110, Seattle, WA USA; 2 Division of Immunology, Boston Children’s Hospital, Harvard Medical School, Boston, MA USA; 3 Institutional Centers for Clinical and Translational Research, Boston Children’s Hospital, Boston, MA USA; 4 Department of Pediatrics Quality Program, Boston Children’s Hospital, Harvard Medical School, Boston, MA USA

**Keywords:** Transition, Transfer of care, Adolescent, Patient-reported outcomes, Survey

## Abstract

Adolescents and young adults (AYA) with rheumatologic diseases are at high risk for poor outcomes and gaps in care when transitioning from pediatric to adult care. However, tools for evaluating transition readiness and assessing the impact of transition interventions are limited. We implemented a written transition policy at our pediatric rheumatology center and evaluated preparation for transition among AYA 16 and older before and after distribution. 31 of 77 patients completed the follow-up survey (response rate 40%). Patient report of transition counseling increased following written transition policy implementation, though these results were not statistically significant in our small cohort. Most follow-up respondents (n = 19, 61%) had not yet completed care transfer; 4 (13%) had arranged a visit with an adult rheumatologist and 8 (26%) had fully transitioned to adult care. Those who successfully completed care transfer were older, had completed higher levels of education, and had significantly higher baseline transition preparation scores compared to those with no transfer arranged or planned visit only. Our single-center pilot study demonstrated that longitudinal assessment of transition preparation is feasible and that scores are significantly associated with care transfer outcomes. Tracking transition preparation over time may provide practices with information on areas of highest need for transition guidance and predict successful transfer among AYA with rheumatologic disease.

## Background

Adolescents and young adults (AYA) are at high risk for poor outcomes and gaps in care when transitioning from pediatric to adult care [1, 2]. The risks of this care transition period are well described in AYA with rheumatologic diseases, including increased disease flares, missed follow up visits, medication non-adherence, and even increased mortality [3–6]. There are increasing efforts to bring comprehensive transition care to AYA with rheumatologic diseases [7–11], though many pediatric rheumatology programs lack formalized transition pathways [12, 13]. Despite increased attention to high-quality transition care, measuring preparation for transition and evaluating transfer outcomes remain challenging. While several instruments have been developed to evaluate AYA preparation for and/or readiness for transition to adult care [14–17], these have not been previously correlated with transfer outcomes among youth with rheumatologic diseases. Additionally, owing to the difficulty of assessing effective transition, many interventions have been evaluated with measures of patient or provider satisfaction rather than objective measures of successful transfer [7, 8], especially in care settings where pediatric and adult specialists are not part of the same integrated healthcare organization.

We evaluated preparation for transition with a validated quality measure prior to implementing a written transition policy at our pediatric rheumatology center, as previously reported in this journal [18]. Following successful dissemination of the written transition policy, we repeated the survey to assess longitudinal changes in transition preparation after implementing the transition policy, and to evaluate the association of transition preparation scores with transfer outcomes.

## Findings

### Methods

We surveyed AYA 16 and older who had an ICD-9 or -10-confirmed rheumatologic diagnosis and had attended ≥3 rheumatology appointments with the ADolescent Assessment of Preparation for Transition (ADAPT) [14] through a REDCap [19, 20] form distributed via email prior to transition policy implementation. This instrument assesses transition preparation based on patient self-report of skills reviewed at the most recent visit (maximum 12 months prior) in three domains: (1) self-management, (2) medications and (3) transfer planning. We created a total composite score comprised of the proportion of affirmative answers to summarize overall preparation and to assess longitudinal change in preparation [18].

**Fig. 1:**
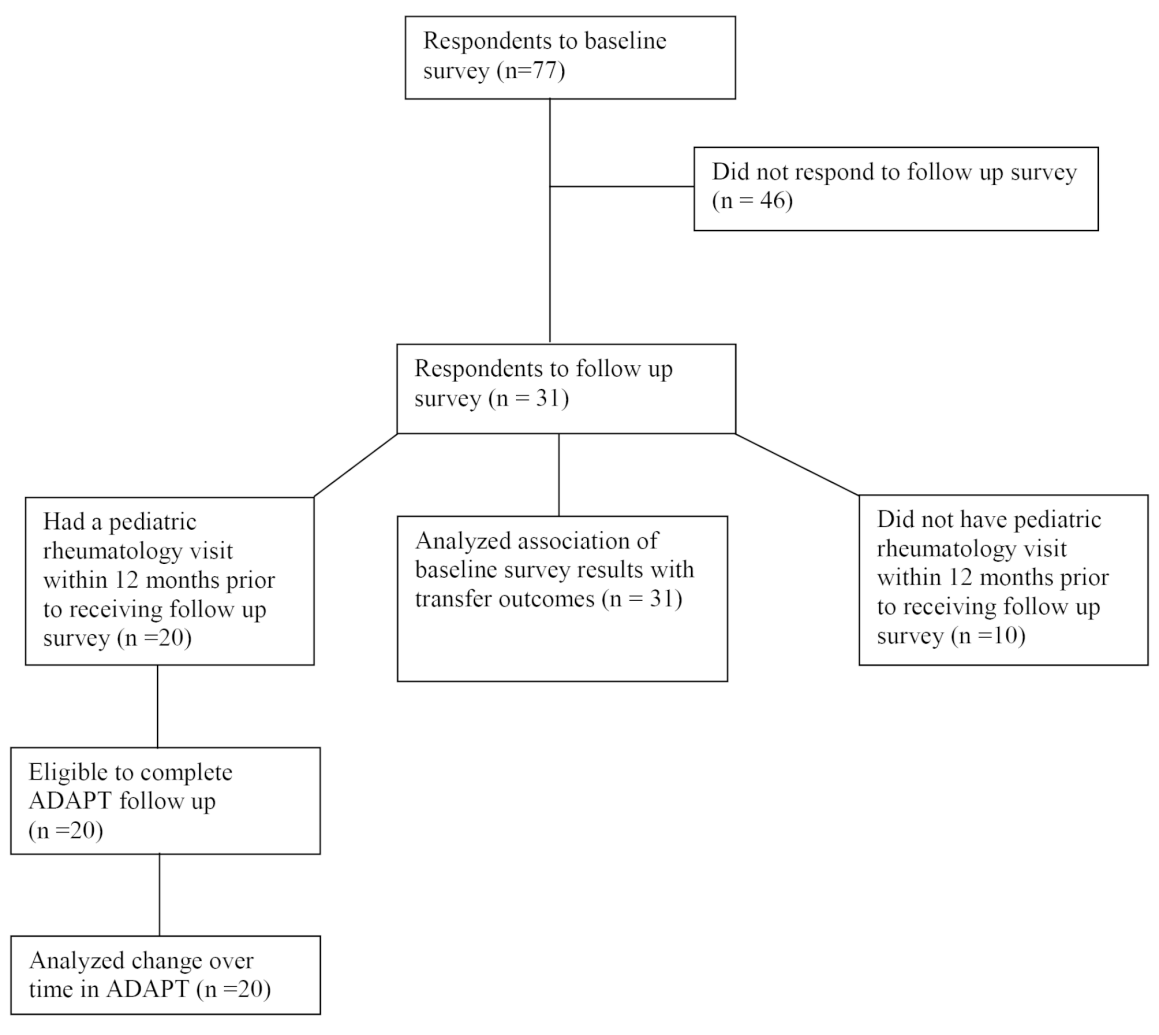
Flow Diagram of Survey Participants

Due to reduction of in-person visits amid the COVID-19 pandemic, in-person distribution of the transition policy at each outpatient visit for all patients 14 and older began approximately 1 year after the initial survey. Patients and parents were asked to sign and return the written policy to confirm dissemination to the intended population. The policy was also made available on our clinic website for patients attending virtual visits. Respondents were invited via email to complete a REDCap follow-up survey including the ADAPT instrument and assessment of care transfer outcomes one year after transition policy implementation, and two years after the baseline survey. Those with a pediatric rheumatology visit within the last 12 months were eligible to complete the ADAPT instrument. Care transfer outcomes, specifically scheduling and attending a visit with an adult rheumatologist, were collected for all respondents regardless of timing of last pediatric rheumatology visit. A flow diagram of participants included in each analysis is presented in Fig. 1. The association between baseline transition preparation scores and transfer outcomes was evaluated using one-way ANOVA. Change in transition preparation over time was assessed with generalized estimating equations (identity or logit link, depending on the outcome) and robust standard errors to account for within patient correlation of repeated measures. This study was approved by the Boston Children’s Hospital Institutional Review Board, protocol number #P00031635.

## Results

Seventy-seven patients participated in our baseline assessment of transition preparation and were eligible for the follow-up survey, of whom 31 responded (40%). Of these, 20 had attended a pediatric rheumatology visit within the last 12 months and therefore were eligible to complete the follow-up ADAPT instrument. Each group of respondents had similar demographic and disease characteristics (Table 1), though owing to the older ages at follow up, respondents reported higher levels of formal education and longer length of time seeing their doctors (p < 0.001) compared to baseline. Respondents to the follow-up survey were more likely to be female compared to baseline respondents (p = 0.045).

**Table 1:** Survey Participant Characteristics by Survey Response

	Respondents to Baseline Survey (n = 77)		Respondents to Follow-Up Survey (n = 31)	Respondents to Follow-Up Survey with Visit in Last Year (n = 20)
	**n (%) or Median [IQR]**			
Race				
White	66 (86)	25 (81)		17 (85)
Black or African American	8 (10)	2 (6)		0 (0)
Asian	1 (1)	1 (3)		1 (5)
Ethnicity				
Not Hispanic, Latino, or Spanish Origin	72 (94)	29 (94)		19 (95)
Puerto Rican	3 (4)	2 (6)		1 (5)
Other Hispanic, Latino, or Spanish Origin	2 (3)	0 (0)		0 (0)
Female	64 (83)	29 (94)		18 (90)
Age (years)	19 [17–20]	20 [20–22]		20 [20–22]
Education				
9th grade	2 (3)	0 (0)		0 (0)
10th grade	9 (12)	0 (0)		0 (0)
11th grade	13 (17)	0 (0)		0 (0)
12th grade, high school graduate, or GED	13 (17)	6 (19)		5 (25)
Some college	33 (43)	14 (45)		10 (50)
College graduate	7 (9)	11 (35)		5 (25)
Primary Rheumatologic Diagnosis*				
Juvenile Idiopathic Arthritis	52 (68)	22 (71)		15 (75)
Uveitis (idiopathic)	2 (3)	1 (3)		0 (0)
Chronic Recurrent Multifocal Osteomyelitis	3 (4)	0 (0)		0 (0)
Systemic Lupus Erythematosus	8 (10)	3 (10)		1 (5)
Mixed Connective Tissue Disease	4 (5)	2 (6)		1 (5)
Sjögren’s	3 (4)	1 (3)		1 (5)
Juvenile dermatomyositis	3 (4)	2 (6)		2 (10)
Other vasculitis	4 (5)	0 (0)		0 (0)
Length of time seeing doctor				
At least 6 months but less than 1 year	2 (3)	0 (0)		0 (0)
At least 1 year but less than 3 years	20 (26)	2 (6)		2 (10)
At least 3 years but less than 5 years	19 (25)	11 (35)		5 (25)
5 years or more	36 (47)	18 (58)		13 (65)
Frequency of visits in past year				
None	0 (0)	11 (35)		0 (0)
1 time	17 (22)	7 (23)		7 (35)
2 times	24 (31)	6 (19)		6 (30)
3 times	17 (22)	4 (13)		4 (20)
4 times	13 (17)	3 (10)		3 (15)
5 or more times	6 (8)	0 (0)		0 (0)
Health status				
Excellent	10 (13)	3 (10)		1 (5)
Very good	18 (23)	8 (26)		7 (35)
Good	32 (42)	16 (52)		11 (55)
Fair	15 (19)	4 (13)		1 (1)
Poor	2 (3)	0 (0)		0 (0)

^*^Some patients have more than 1 primary rheumatologic diagnosis.

Scores in the self-management and medication counseling domains and total composite scores at follow-up showed small numerical increases compared to the baseline, though this did not reach statistical significance in our small cohort (Table 2). In contrast, the median transfer planning score did not differ over time, with few respondents answering affirmatively to questions about discussing a specific transfer plan and receiving this plan in writing.

**Table 2:** Change Over Time in Selected ADAPT Transition Preparation Measures

	Baseline Survey N = 77	Two Year Follow-Up N = 20	p-value
**Survey Question**	**Yes** **n (%)**	**Yes** **n (%)**	
In the last 12 months, did you talk with this provider without your parent or guardian in the room?	46 (59.7%)	12 (60.0%)	0.50
In the last 12 months, did you and this provider talk about you being more in charge of your health?	53 (68.8%)	14 (70.0%)	0.91
In the last 12 months, did you and this provider talk about remembering to take your medicines?	37 (75.5%)	8 (80.0%)	0.49
In the last 12 months, did you and this provider talk about whether you may need to change to a new provider who treats mostly adults?	31 (40.8%)	8 (44.4%)	0.68
In the last 12 months, did this provider ask if you had any questions or concerns about changing to a new provider who treats mostly adults?	22 (71.0%)	6 (75.0%)	0.82
In the last 12 months, did you and this provider talk about a specific plan for changing to a new provider who treats mostly adults?	14 (45.2%)	5 (62.5%)	0.22
**Composite Transition Preparation Scores**	**Median [IQR]**	**Median [IQR]**	
Self-management (highest score possible 4)	2 [1–3]	2.5 [1–3]	0.36
Medication counseling (highest score possible 3)	2 [2,3]	2.5 [2,3]	0.58
Transfer planning (highest score possible 4)	0 [0–2]	0 [0–3]	0.31
Total transition composite score (% of items answered yes)	0.45 [0.27–0.55]	0.52 [0.32–0.64]	0.26

At the time of follow-up survey, most respondents (n = 19, 61%) had not yet completed care transfer to adult rheumatology (Table 3). Four (13%) had arranged a visit with an adult rheumatologist and 8 (26%) had fully transitioned to adult care. Those who had successfully completed rheumatology care transfer were older and had completed higher levels of education compared to those with no transfer arranged or planned visit only. Respondents with higher baseline transition preparation scores were significantly more likely to have completed care transfer to an adult rheumatologist.

**Table 3:** Patient Characteristics and ADAPT Scores by Transfer Outcome

	Completed Transfer	Visit Arranged	No Transfer Arranged	p-value
	**n = 8**	**n = 4**	**n = 19**	
Age	22.5 [21–23]	22 [21–22.5]	20 [19–21]	0.009
Education Level				0.007
High School Graduate	0 (0)	0 (0)	6 (32)	
Some College	2 (25)	1 (25)	11 (58)	
College Graduate	6 (75)	3 (75)	2 (11)	
Female	8 (100)	4 (100)	17 (89)	0.51
Primary Rheumatologic Disease				0.29
Juvenile Idiopathic Arthritis	4 (50)	4 (100)	14 (74)	
Idiopathic Uveitis	0 (0)	0 (0)	1 (5)	
Systemic Lupus Erythematosus	2 (25)	0 (0)	1 (5)	
Mixed Connective Tissue Disease	2 (25)	0 (0)	0 (0)	
Sjögren’s	0 (0)	0 (0)	1 (5)	
Juvenile dermatomyositis	0 (0)	0 (0)	2 (11)	
**Composite scores: Baseline**	**n = 8**	**n = 4**	**n = 19**	
Total transition composite score (% yes)	0.68 [0.48–0.88]	0.52 [0.44–0.64]	0.45 [0.27–0.55]	0.01
Self-management (highest possible 4)	3 [2-3.5]	2.5 [1-2]	3 [1-3]	0.36
Transfer planning (highest possible 4)	3 [0.5–3.5]	1.5 [0.5-3]	0 [0–1]	0.005
	**n = 4**	**n = 2**	**n = 14**	
Medication counseling (highest possible 3)	2.5 [1.5-3]	3 [3]	2 [2,3]	0.29
Composite scores: Follow Up*	**n = 1**	**n = 2**	**n = 17**	
Total transition composite score	0.64	0.56 [0.12-1.00]	0.50 [0.36–0.64]	0.80
Self-management	3	2.5 [1–4]	2 [1–3]	0.82
Transfer planning	3	2 [0–4]	0 [0–2]	0.37
	**n = 1**	**n = 1**	**n = 8**	
Medication counseling	1	3	2.5 [2, 3]	0.20

^*^Only patients with last rheumatology visit within 12 months were eligible to complete follow-up ADAPT (n = 20). Medication and transfer composites applicable only to those patients screening in based on earlier questions

## Conclusion

In this single center pilot study, we demonstrated the feasibility of tracking patient preparation for transition longitudinally to evaluate change over time and assess response to a transition intervention, which was the distribution of a clinic transition policy in this report. We did not find significant improvements in preparation scores between the pre-policy and post-policy periods, which may be due to the limited impact of the transition policy intervention, or to underpowering given the low numbers of eligible respondents in the follow-up survey.

We found that baseline transition preparation scores were associated with patient-reported transfer outcomes, including attendance at a visit with an adult rheumatologist. By demonstrating the association of transition preparation scores with transfer outcomes in youth with rheumatologic diseases, our findings may identify at-risk AYA in need of additional support during the transition period, and to predict patients who may be ready for successful transfer. Tracking transition preparation longitudinally may provide practices with information on areas of highest need for transition counseling. For example, in our cohort we saw lowest scores in the transfer planning domain over both time points, which highlights the need to incorporate more focused discussions on these topics into clinical care.

Our study had several limitations, including small sample size, and delays in policy implementation due to COVID-19 which lengthened the time interval between the baseline and follow-up surveys, and may have impacted our response rate. COVID-related delays in care may also have impacted the frequency of visits during the follow-up period, decreasing exposure to the written transition policy and limiting opportunities to discuss the policy with the care team, as described by other centers attempting transition policy implementation during the pandemic [21]. Additionally, as a single-center study, our results may not be generalizable to all care settings. Our center is a quaternary care children’s hospital without an institution-wide age of transition and with several highly specialized programs where patients may be followed into adulthood. Age to initiate discussions about transition and transfer have traditionally been left to individual providers’ discretion, which may account for relatively low levels of transition counseling and higher age of transfer among our cohort.

Despite these limitations, our pilot study demonstrated that longitudinal assessment of transition preparation is feasible and predicts successful transfer outcomes. Further research is needed in larger cohorts to validate the association between transition preparation scores and transfer outcomes. Finally, higher transition preparation scores may be a function of both the actual transition counseling received, and patient resources, including self-management skills [22, 23]. Such self-management skills have also been shown to be correlated with socioeconomic status [24], which may itself impact successful transfer [4–6]. Therefore, identifying elements of transition preparation that are most readily modifiable via transition counseling and other clinical team supports may help to design more effective interventions for AYA with rheumatologic diseases who are at risk of poor transfer outcomes.

## Data Availability

The datasets generated and analyzed during the current study are available from the corresponding author on reasonable request and pending approval of data sharing by the Boston Children’s Hospital IRB.

## Abbreviations

ADAPT: ADolescent Assessment of Preparation for Transition.
AYA: adolescents and young adults.
